# Oxidative stress and DNA damage in patients with migraine

**DOI:** 10.1186/s10194-016-0606-0

**Published:** 2016-02-17

**Authors:** Sırma Geyik, Erman Altunısık, Ayse Münife Neyal, Seyithan Taysi

**Affiliations:** Department of Neurology, Gaziantep University, Gaziantep, Turkey; Division Of Neurology, Turkish Ministry of Health Siirt State Hospital, Siirt, Turkey; Department of Biochemistry, Gaziantep University, Gaziantep, Turkey; Department of Neurology, Faculty of Medicine, University of Gaziantep, Gaziantep, 27130 Turkey

**Keywords:** Migraine, Oxidative damage, Oxidative DNA damage, 8-hydroxydeoxyguanosine

## Abstract

**Background:**

Oxidative stress is implicated in the pathogenesis of migraine, but no published studies have examined both oxidative stress levels and oxidative DNA damage on the same patient group.

**Methods:**

In this study, total oxidant status (TOS); total antioxidant status (TAS); oxidative stress index (OSI); and 8-hydroxy-2′-deoxyguanosine (8-OHdG), which is an indicator of oxidative DNA damage, were measured in the plasma samples of 50 prophylactic unmediated migraineurs (11 with aura and 39 without aura) and 30 matched healthy volunteers.

**Results:**

No significant differences in TAS, TOS, and OSI values were observed between patients and controls. However, plasma 8-OHdG levels were found to be significantly higher in migraine patients than in the control group (*p* = 0.001); this increase in plasma 8-OHdG levels was more prominent in cases with migraine without aura than with aura (*p* = 0.001). Our results suggested an evidence of oxidative stress-related DNA damage in migraine.

**Conclusion:**

DNA damage reflected by plasma 8-OHdG did not studied in migraine before. Therefore, further research on oxidative stress-related DNA damage and the extent of its clinical manifestations in migraine may provide additional data to our current knowledge.

## Background

Oxidative stress, which arises because of an imbalance between the production of reactive oxygen species (ROS) and elimination by antioxidant defense mechanisms, has been implicated in various headache disorders [1–3] including migraine [4, 5]. Endogenous ROS can cause oxidative damage to DNA, lipids, and proteins [6]. Oxidants may also confer susceptibility to other pathogenic processes by disrupting the functions of cytoprotective proteins, such as metabolic enzymes and cell membrane transporters [7]. Among the DNA constituents, guanine has the lowest ionization potential; therefore, it is highly susceptible to the actions of free radicals. The oxidation product, 8-hydroxy-2′-deoxyguanosine (8-OHdG) generated by the hydroxylation of guanine at the C8 position is one of the most common biomarkers used to detect oxidative DNA base damage induced by ROS [8]. It is also used as a marker of oxidative stress, mitochondrial dysfunction, and impaired metabolism [9]. Subsequent to oxidation, damaged DNA is repaired by cellular mechanisms and hydroxylated guanine is eliminated in body fluids [8, 10].

Many studies have been conducted to elucidate the pathophysiology of migraine. However, it is currently not possible to explain the etiopathogenesis of the disease by a single theory. Recent studies supported the potential role of oxidative stress and related structures [11–13], but, to the best of our knowledge, no published study has simultaneously investigated oxidative stress parameters and oxidative DNA damage indicators on the same group of patients with migraine.

In the present study, we aimed to evaluate the plasma total oxidant status (TOS); plasma total antioxidant status (TAS); oxidative stress index (OSI); and 8-hydroxy-2′-deoxyguanosine (8-OHdG), which is an indicator of oxidative DNA damage, in patients with migraine in the interictal phase, taking into account the different subgroups of patients with and without aura.

We assessed the intergroup differences and the effects of demographic, clinical, and laboratory characteristics on the subgroups of migraine associated with aura and without aura.

## Methods

### Study population

Study approval was obtained from the Ethics Committee of Gaziantep University Faculty of Medicine (24.3.2014/106). Informed consent was obtained from all subjects prior to the study. This study examined 50 consecutive patients aged 18–45 years, who were diagnosed to have migraine and were not on prophylactic treatment for at least 3 months and for at least 72 h symtomathic drug-free period before obtaining blood samples in the interictal phase. Migraine, both with and without aura, was diagnosed according to the ICHD-3 beta criteria [14] by a single experienced neurologist in our clinic and then enrolled to the study. The control group (*n* = 30) was composed of healthy health care workers and medical students who worked in our hospital, met the criteria for joining the study, signed informed consent form and matched with the patient group with respect to age and sex. Cases with moderate or severe mental retardation; history of significant head trauma; malnutrition; pregnancy; diabetes mellitus (fasting blood glucose ≥ 120 mg/dl); hypertension (BP ≥ 140/90 mmHg); chronic renal failure; liver cirrhosis; any type of cancer; thyroid diseases; alcohol and substance abuse; chronic neurologic illnesses, including epilepsy, Parkinson’s disease, Huntington’s disease, Alzheimer’s disease, Wilson’s disease, and previous cerebrovascular disease; morbid obesity; and any existing infection were excluded from both groups. Other exclusion criteria were the use of glucocorticoids; oral contraceptives; antioxidant agents, such as vitamin E, vitamin C, or N-acetylcysteine; and xanthine oxidase inhibitors, such as allopurinol or folic acid.

The medical history, physical and neurologic examination findings, and body mass index (BMI) of all cases were recorded on a previously structured form. The migraine characteristics, such as type, frequency within the last 3 months, associated features, etc., were also recorded for the migraine group. Routine laboratory examinations, including total blood count, serum electrolytes, serum creatinine, blood urea nitrogen (BUN), fasting blood glucose levels, and liver function tests, were performed in all cases. The Migraine Disability Assessment Scale (MIDAS) was used to assess the severity of migraine in the migraine group.

### Blood sampling

Venous blood samples were drawn from the antecubital vein in the morning hours after 12 h of fasting and for at least 72 h without symtomethic migraine medication. Plasma was separated from blood samples into plain biochemistry tubes by centrifugation at 3000 rpm for 5 min at 4 °C. Plasma samples were stored at −80 °C until the measurement of total antioxidant status (TAS), total oxidant status (TOS), and 8-OHdG.

### Measurement of study variables

Plasma TAS and TOS were measured by a fully automated colorimetric assay developed by Erel [15, 16]. Fe^2+^–o-dianisidine complex gave a Fenton-type reaction with hydrogen peroxide to form the OH radical. This powerful ROS reacted with the colorless o-dianisidine molecule at a reducing low pH to form yellow–brown dianisidyl radicals. Dianisidyl radicals participated in further oxidation reactions, resulting in a more color formation. The antioxidants in the samples suppressed these oxidation reactions and inhibited color formation; this reaction was measured spectrophotometrically by automatic analyzers. According to this method thiol groups are responsible 52.9 % and uric asit is responsible 33.1 % of the total antioxidant capacity of healthy individuals [17]. The oxidants in the sample converted the Fe^2+^–o-dianisidine complex to ferric ion; glycerol in the media accelerated this reaction by about 3-fold. The ferric ions formed a colored compound with xylenol orange in the acidic media; this color was associated with the amount of oxidants in the sample and was spectrophotometrically measured. In this method Total Oxidant Status refers mostly to plasma levels of hydrogen peroxide and lipid hydroperoxides. [16].

The ratio of plasma TOS to TAS levels was determined as the oxidative stress index (OSI). Serum levels of 8-OHdG were measured using the Northwest kit (NWLSS 8-OHdG ELISA High Sensitivity kit, Northwest; Vancouver, Canada), a competitive ELISA kit suitable for the quantification of DNA oxidation in tissues, serum, and plasma.

### Statistical analyses

The Kolmogorov–Smirnov test was used to check whether continuous variables conformed to normal distribution. Student’s *t*-test was used for the pair-wise comparison of normally distributed data sets. The Mann–Whitney *U* test was used for the pair-wise comparison of non-normally distributed datasets. Categorical variables were compared using the chi-square test and the coefficient of correlation was used to test the strength of association between numerical variables. Values were expressed as mean ± standard deviation (SD). SPSS for Windows version 22.0 (IBM Corp., USA) was used for statistical analyses and *p* < 0.05 was considered statistically significant.

## Results

The demographic characteristics of the study population are shown in Table 1. The patient and control groups did not significantly differ in sex, mean age, mean BMI, and smoking status (*p* > 0.05). Twenty-six (52 %) migraine patients were using symptomatic drugs (triptans, NSAID, paracetamol and combination analgesic). Out of the 50 migraine cases, 11 had MWA and 39 had MWoA. The subgroup comparison of the patient group is presented in Table 2. There were no significant differences in the frequency of migraine attacks, duration of disease, age at onset, and MIDAS scores between the MWA and MWoA groups (*p* > 0.05).

**Table 1:** Demographic characteristics of the study population

Parameter	Controls (*n* = 30)	Patients (*n* = 50)	*p*
Mean age (years)	29.60 ± 5.46	31.78 ± 5.81	0.101
Gender (*n*, %)			
Female	19 (63.3)	36 (72.0)	0.418
Male	11 (36.7)	14 (28.0)	
BMI (kg/m^2^)	23.19 ± 2.37	22.63 ± 2.74	0.357
Smoking (*n*, %)	18 (60.0)	35 (70.0)	0.360

*BMI* body mass index

**Table 2:** Subgroup analysis of patients with migraine

Parameter	MWA (*n* = 11)	MWoA (*n* = 39)	*p* value
Age at disease onset (years)	23.58 ± 4.2	21.12 ± 5.6	0.240
Duration of disease (years)	9.72 ± 6.29	7.66 ± 4.85	0.249
Frequency of attacks (within the last 3 months)	2.18 ± 0.98	4.51 ± 4.04	0.363
Vomiting during attacks (*n*, %)	4 (36.4)	22 (56.4)	0.240
Mood changes (*n*, %)	10 (90.9)	37 (94.9)	0.625
MİDAS score	2.36 ± 1.20	2.92 ± 1.10	0.146

*MWA* migraine with aura, *MWoA* migraine without aura, *MIDAS* Migraine Disability Assessment Scale

**Fig. 1:**
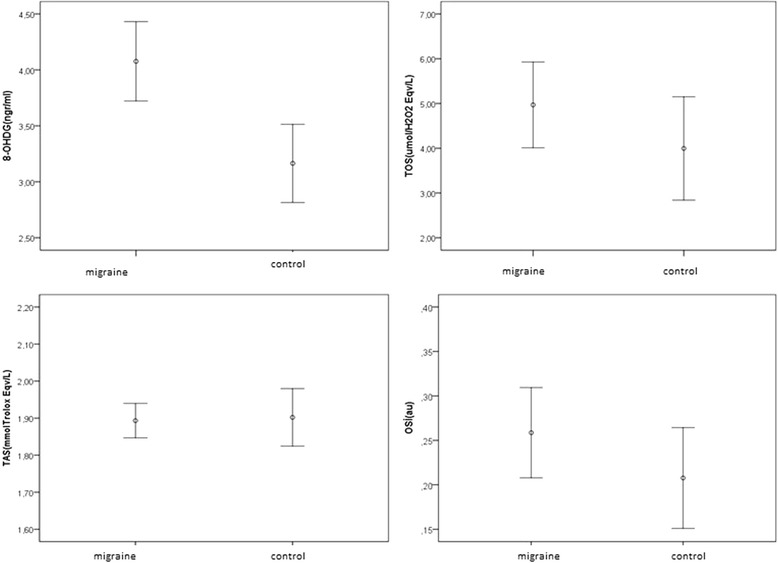
Serum concentrations of 8-OHdG, TAS, TOS and OSİ in migraine and healthy control groups

Migraineurs showed no significant differences in TAS, TOS, and OSI values compared with the controls (*p* > 0.05) (Table 3). However, serum 8-OHdG was significantly higher in patients with migraine than in healthy controls (*p* = 0.001) (Table 3) (Fig. 1). TAS, TOS, and OSI did not significantly differ between the MWA and MWoA patient subgroups (*p* > 0.05), but plasma 8-OHdG was significantly higher in MWoA patients (*p* = 0.001) (Table 4).

**Table 3:** Analysis of oxidative stress parameters in patients with migraine

Parameter	Patients (*n* = 50)	Controls (*n* = 30)	*p* value
TAS (mmolTroloxEqv/L) (mean ± SD)	1.89 ± 0.16	1.90 ± 0.20	0.862
TOS (mmolTroloxEqv/L) (mean ± SD)	4.96 ± 3.37	3.99 ± 3.09	0.080
OSI (mean ± SD)	0.25 ± 0.17	0.20 ± 0.15	0.110
8-OHdG (ng/mL) (mean ± SD)	4.07 ± 1.24	3.16 ± 0.93	0.001^*^

Values are expressed as mean ± SD; *TAS* total antioxidant status, *TOS* total oxidant status, *OSI* oxidative stress index, *8*-*OHdG* 8-hydroxy-2′-deoxyguanosine

*SD* Standard deviation *p < 0.05

**Table 4:** Comparison of oxidative stress parameters according to the type of migraine

Parameter	MWA (*n* = 11)	MWoA (*n* = 39)	*p* value
TAS (mmolTroloxEqv/L) (mean ± SD)	1.87 ± 0.17	1.89 ± 0.16	0.582
TOS (mmolTroloxEqv/L) (mean ± SD)	4.76 ± 4.29	5.02 ± 3.13	0.178
OSI (mean ± SD)	0.25 ± 0.22	0.26 ± 0.16	0.313
8-OHdG (ng/mL) (mean ± SD)	3.11 ± 0.65	4.34 ± 1.24	0.001^*^

Values are expressed as mean ± SD

*MWA* migraine with aura, *MWoA* migraine without aura, *TAS* total antioxidant status, *TOS* total oxidant status, *OSI* oxidative stress index, *8*-*OHdG* 8-hydroxy-2′-deoxyguanosine, *SD* Standard deviation *p < 0.05

TAS and TOS levels did not differ by gender, by smoking status or by symptomatic medication although 8-OHdG was significantly higher in patient group than healty group accordingly all of these variables (Table 5).

**Table 5:** Comparison of oxidative stress parameters according to demographic variables

Demographic variables	TAS (mmolTroloxEqv/L)		*p* value	TOS (mmolTroloxEqv/L)		*p* value	8-OHdG (ng/mL)		*p* value
	Migraine	Control (*n* = 35)		Migraine	Control		Migraine	Control	
Smoking	1.88 ± 0.45	1.82 ± 0.52	0.798	5.21 ± 3.8	4.06 ± 3.4	0.091	4.12 ± 1.2	3.31 ± 1.8	0.001*
Non-smoking	1.9 ± 0.89	1.94 ± 0.7	0.892	4.86 ± 3.2	3.94 ± 3.1	0.064	3.96 ± 1.08	2.98 ± 1.2	0.001*
*p* value	0.639	0.017		0.768	0.518		0.465	0.662	
Female	1.7 ± 0.38	1.88 ± 0.62	0.164	4.82 ± 3.9	3.86 ± 2.9	0.065	3.92 ± 1.3	2.92 ± 1.8	0.001*
Male	1.9 ± 0.45	1.92 ± 0.48	0.692	4.99 ± 3.4	4.01 ± 3.7	0.074	4.19 ± 1.1	3.38 ± 1.4	0.001*
*p* value	0.598	0.765		0.794	0.684		0.235	0.085	
Drugs (+)	1.93 ± 0.17	–		5.48 ± 3.8	–		4.16 ± 1.2	–	
Drugs (−)	1.85 ± 0.14	–		4.49 ± 2.8	–		3.99 ± 3.2	–	
*p* value	0.103			0.290			0.336		

Values are expressed as mean ± SD; *TAS* total antioxidant status, *TOS* total oxidant status, *OSI* oxidative stress ındex, *8*-*OHdG* 8-hydroxy-2′-deoxyguanosine, *SD* standard deviation *p < 0.05

Drug (+): Using symtometic migraine drugs (*n* = 24)

Drug (−): Not using symtometic migraine drugs (*n* = 26)

**Fig. 2:**
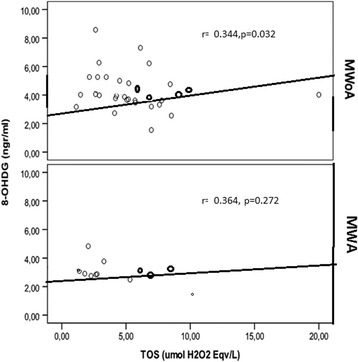
Correlation between 8-OHdG and TOS in MWA and MWoA

Disease duration or frequency of attacks and MIDAS score were not significantly correlated with TAS, TOS, OSI and 8-OHdG levels (Table 6). For the entire migraine group, there was no significant correlation among 8-OHdG levels and TOS, TAS, OSI; whereas in the subgroup comparison, 8-OHdG levels were positively correlated with TOS and OSI in the MWoA group (*r* = 0.344, *p* = 0.032) (Fig. 2).

**Table 6:** Correlation analysis between migraine characteristics and plasma indicators of oxidative stress

	OHDG	TAS	TOS	OSİ
	(r/p)	(r/p)	(r/p)	(r/p)
Disease duration	0.45/0.757	−0.275/0.053	0.152/0.293	−0.198/0.167
Frequency	0.195/0.175	−0.221/0.123	0.53/0.716	−0.42/0.776
MIDAS	0.204/0.154	−0.197/0.170	0.36/0.804	−0.046/0.753

Spearman’s rho correlation analysis was performed

## Discussion

The present study revealed significantly high plasma levels of 8-OHdG, an indicator of oxygen radical-induced DNA damage, in migraine patients, which was more prominent in MWoA cases. However, plasma TOS and TAS levels, and OSI of migraine patients were statistically similar to that of the control group.

Migraine is defined as a neurovascular disorder that involves spreading cortical depression, neurogenic inflammation, and dysfunction in cranial vascular contractility [18]. Noxious free radicals produced as a result of metabolic and physiologic processes are normally neutralized by enzymatic and non-enzymatic antioxidant systems. The balance can shift towards a state of oxidative stress due to an increased production of free radicals or a deficiency in antioxidant defense mechanisms [15]. Oxidative stress can damage membrane lipids, nucleic acids, proteins, and extracellular matrix components, including proteoglycans and collagens [19]. Plasma 8-OHdG reflects oxidative damage induced by free radicals to nuclear and mitochondrial DNA [8].

The role of oxidative stress in migraine was discussed in various studies [4, 20, 21]. In Eren et al. study, no difference was found in TAS, TOS, OSI between the migraine patients and controls. The level of thiol which created the 52.9 % of TAS was significantly lower in patients than in controls. The results of our present study are compatible with this study in terms of TAS, TOS and OSİ. [5], whereas contradictory the results of Alp et al. study in which they suggested that the levels of TAS were decreased and the levels of TOS and OSİ were increased in patients with MWoA [20]. These conflicting results may be because of the various techniques used, the biological samples analyzed, timing of sampling, and differences in subject selection.

Both TOS and TAS have been shown to be reliable and sensitive indicators of the current oxidant-antioxidant situation in the body at a given time. However, similar to the others that are used to evaluate oxidative stress status, both of these parameters may vary according to factors, such as used technique, sampling hours in the day, physiologic and pathologic events in the body, current drug intake, patient habits, etc. Therefore, it is not easy to claim that all of the findings regarding the oxidative stress parameters are merely linked to the specific disease that is in the interest of research in this area. This is true even for the studies that were designed in the consideration of all parameters mentioned above, as was in this study. Further, we believe that it would be more difficult to make such recommendations for cases of paroxysmal disorders such as migraine. Even if the phase of migraine (with or without attack) is controlled in a study design, researchers may not be able to completely homogenize the study population. On the other hand, the consequences of oxidative stress, even in paroxysmal disorders, may be more easily and stably detected in a wider time interval. This point of view may possibly explain our results. Although to decrease this possibility we preferred to take the blood samples in the interictal phase from all of the patients. Smoking, sex, symptomatic medication use are other factors that can affect the oxidative stress status. Higher levels of MDA, 8-OHdG, SOD and GSH-Px have been shown in smokers [22]. Bloomer et al. suggested that oxidative stress was lower in women than in men [23]. Orhan et al. suggested that NSAIDs and paracetamol may involve in oxidative/antioxidative processes in the body directly or indirectly [24]. Therefore to minimize these effects, variables were matched in both groups and the blood samples were taken for at least 72 h drug-free period. In our results smoking, sex and symptomatic medication use did not affect the comprasion of TOS, TAS and 8-OHdG levels between groups.

Increased levels of 8-OHdG have been shown in studies on oxidative stress-related disorders [25–27], but the exact biological role of 8-OHdG has been still unknown. In this study, confounding factors that determine natural antioxidant enzyme capacity, such as SOD, were not accounted for and could have affected the serum 8-OHdG levels. Nevertheless, the similar TAS levels in both our study groups revealed that total antioxidant capacity was not defective in migraine cases. Altought plasma thiol groups might not adequately reflect redox status in other tissues, such as the brain [28].

One interesting point in the present study was that the plasma 8-OHdG levels were significantly higher in MWoA than in MWA. Although there were no significant differences in terms of clinical characteristics of the subgroups, the frequency of attacks and MIDAS scores were higher in MWoA patients. Therefore, higher levels of 8-OHdG may be related to the more severe and frequent attacks but not to the type of migraine.

The relatively small number of patients in both migraine groups, particularly in the MWA group, limits the reliability of oxidative stress parameters comparison between patients with or without aura. Our results should be verified by further studies. We think these are just preliminary results. Nevertheless, all biochemical assessments of TOS, TAS, and OSI were done in the same session by experienced experts in the same laboratory. Therefore, the probability of technical error was low in the present study. However, because there have been no other studies that had a similar design to ours, we cannot claim that TAS and TOS plasma levels and OSI are the most suitable indicators of the relationship between oxidative stress and its consequences, such as DNA damage.

## Conclusion

Therefore, further research on oxidative stress-related DNA damage and the extent of its clinical manifestations in migraine may provide additional data to our current knowledge.

## Abbreviations

TOS: total oxidant status
TAS: total antioxidant status
OSI: oxidative stress index
8-OHdG: 8-hydroxy-2′-deoxyguanosine
ICHD-3 beta: International Classification of Headache Disorders 3rd edition-beta version
MWA: migraine with aura
MWoA: migraine without aura
ROS: reactive oxygen species
BP: blood pressure
MIDAS: Migraine Disability Assessment Scale
MRI: Cranial magnetic resonance imaging
CT: computed tomography
BMI: body mass index
SD: standard deviation
SOD: superoxide dismutase
GSH-Px: glutathione reductase
GR: glutathione reductase
CAT: catalase
